# FBXW7-mediated ERK3 degradation regulates the proliferation of lung cancer cells

**DOI:** 10.1038/s12276-021-00721-9

**Published:** 2022-01-12

**Authors:** Hyun-Jung An, Cheol-Jung Lee, Ga-Eun Lee, Youngwon Choi, Dohyun Jeung, Weidong Chen, Hye Suk Lee, Han Chang Kang, Joo Young Lee, Dae Joon Kim, Jin-Sung Choi, Eun Suh Cho, Jong-Soon Choi, Yong-Yeon Cho

**Affiliations:** 1 College of Pharmacy, The Catholic University of Korea, 43, Jibong-Ro, Wonmi-Gu, Bucheon-si, Gyeonggi-Do 14662 Republic of Korea; 2 BK21-4th, and BRL, College of Pharmacy, The Catholic University of Korea, 43, Jibong-Ro, Wonmi-Gu, Bucheon-Si, Gyeonggi-Do 14662 Republic of Korea; 3 Research Center for Materials Analysis, Korea Basic Science Institute, 169-148, Gwahak-Ro, Yuseong-Gu, Daejeon, 34133 Republic of Korea; 4 Department of Immunology and Microbiology, School of Medicine, University of Texas Rio Grande Valley, MBMRF, 1.410, 5300, North L St., McAleen, TX 78504 USA; 5 College of Biological Science, University of Minnesota, 3-104 MCB, 420 Washington Ave SE, Minneapolis, MN 55455 USA; 6 Graduate School of Analytical Science and Technology, Chungnam National University, 99, Daehak-Ro, Yuseong-Gu, Daejeon, 34134 Republic of Korea

**Keywords:** Protein quality control, Ubiquitylation

## Abstract

Extracellular signal-regulated kinase 3 (ERK3) is an atypical member of the mitogen-activated protein kinase (MAPK) family, members of which play essential roles in diverse cellular processes during carcinogenesis, including cell proliferation, differentiation, migration, and invasion. Unlike other MAPKs, ERK3 is an unstable protein with a short half-life. Although deubiquitination of ERK3 has been suggested to regulate the activity, its ubiquitination has not been described in the literature. Here, we report that FBXW7 (F-box and WD repeat domain-containing 7) acts as a ubiquitination E3 ligase for ERK3. Mammalian two-hybrid assay and immunoprecipitation results demonstrated that ERK3 is a novel binding partner of FBXW7. Furthermore, complex formation between ERK3 and the S-phase kinase-associated protein 1 (SKP1)-cullin 1-F-box protein (SCF) E3 ligase resulted in the destabilization of ERK3 via a ubiquitination-mediated proteasomal degradation pathway, and FBXW7 depletion restored ERK3 protein levels by inhibiting this ubiquitination. The interaction between ERK3 and FBXW7 was driven by binding between the C34D of ERK3, especially at Thr417 and Thr421, and the WD40 domain of FBXW7. A double mutant of ERK3 (Thr417 and Thr421 to alanine) abrogated FBXW7-mediated ubiquitination. Importantly, ERK3 knockdown inhibited the proliferation of lung cancer cells by regulating the G_1_/S-phase transition of the cell cycle. These results show that FBXW7-mediated ERK3 destabilization suppresses lung cancer cell proliferation in vitro.

## Cancer: Controling the stability of a pro-proliferation protein

An enzymatic process that marks a cellular signaling protein for destruction may be a check against uncontrolled proliferation in some cancers. Prior studies have indicated that the ERK3 protein can influence essential functions like cell division and migration and that its effects are governed by its stability. Researchers led by Yong-Yeon Cho at The Catholic University of Korea, Bucheon-si, have now determined that an enzyme called FBXW7 controls ERK3 by tagging it with a modification that marks it for destruction. ERK3 has been linked to cancer, although the data have been contradictory as to whether it controls or promotes malignancy. The authors show that ERK3 can promote proliferation in cultured lung cancer cells and that this proliferation can be counteracted by FBXW7. However, ERK3’s general role in tumors is likely to be context-specific.

## Introduction

Extracellular signal-regulated kinase 3 (ERK3) family members are atypical MAPKs and include ERK3, ERK4, ERK7, and Nemo-like kinase (NLK)^1^. The biological activities of these members are mainly regulated by phosphorylation at the activation loop motif Thr-Xaa-Tyr that is catalyzed by a family of dual-specificity protein kinases^2^ and by subcellular localization^3,4^. A structural difference within the highly conserved activation loop motif sequence Ser-Glu-Gly (rather than Thr-Xaa-Tyr) is responsible for the functional characteristics of ERK3 that distinguish it from other MAPKs^5^. Strikingly, unlike other MAPK members, ERK3 is an unstable protein with a half-life of <30 min^5^. Thus, it appears likely that ERK3 expression reflects its activity. Turnover experiments using deletion and truncation mutants of ERK3 have indicated that neither kinase activity nor the C-terminal extension of ERK3 is required for its high turnover^5^. ERK3 harbors two degradation domains in its N-terminal lobe that are both necessary and sufficient to target ERK3 for ubiquitination and degradation via the proteasomal degradation pathway^5,6^. However, the domain(s) of ERK3 that interact with E3 ubiquitin ligase(s) and the identities of the responsible E3 ligase(s) have not been elucidated.

The roles of ERK3 in cancer are controversial. Previous reports have demonstrated that ERK3 overexpression in NIH3T3 cells suppresses BrdU incorporation^7^ and that the expression of stabilized forms of ERK3 causes G_1_ cell cycle arrest in NIH3T3 cells^5^. These results suggest that ERK3 inhibits cell proliferation. However, others have reported that ERK3 expression is upregulated in several human cancer microarrays databases^8–11^ and that ERK3 is upregulated in human lung cancer tissues^12^. However, in an in vitro cell culture system, ERK3 overexpression slightly suppressed HeLa cell proliferation^13^, and in lung cancer cells, ectopic ERK3 expression significantly induced cell proliferation^14^. However, the physiological roles of ERK3 have been studied in the contexts of cell migration and invasion. Recent reports have indicated that ERK3 is required for the maintenance of epithelial architecture via regulation of epithelial morphogenesis and differentiation^15^. In this process, ERK3 plays an essential role in IL-8 production and chemotaxis of leukocytes by modulating c-Jun/AP-1 activity^16^. Moreover, β-adrenergic stimulation stabilizes the ERK3 protein, resulting in an increase in ERK3/MK5 complex formation^17^. This process transduces activation of FOXO1 and promotes the expression of the major lipolytic enzyme ATGL^17^. Moreover, since PKC-mediated ERK3 serine phosphorylation and microtubule-associated protein 2 (MAP2) association are involved in glucose-induced insulin secretion and since ERK3 expression is modulated by prolactin in isolated rat pancreatic islets^18^, ERK3 seems to be involved in insulin secretion^19^. However, the detailed molecular action mechanisms of ERK3, physiological conditions for ERK3 activity regulation, and downstream target proteins, including transcription factors, are not well understood.

Substrate selectivity for F-box-mediated ubiquitination is mediated by two major functional domains: the F-box motif, which directly binds to the adaptor protein SKP1 and recruits the F-box protein into the S-phase kinase-associated protein 1 (SKP1)-cullin 1-F-box protein (SCF) complex, and various carboxyl-terminal domains, which bind to specific substrates^20^. FBXW7 has three isotypes, FBXW7α, FBXW7β, and FBXW7γ, that exhibit different subcellular localizations (i.e., nucleoplasm, cytoplasm, and nucleoli, respectively)^21^. Thus, the isotype specificities of FBXW7 for substrates are dependent on the subcellular localization of FBXW7, its substrates, and cellular signaling. FBXW7 substrates include cyclin E, MYC, JUN, MOTCH, myeloid cell leukemia 1 (MCL1), SREBP, mTOR, KLFs, CCAAT/enhancer-binding proteins (C/EBPs), and the mediator complex components MED13 and MED13L, which all contain a conserved phosphorylated degron sequence, (Leu)-pThe/pSer-Pro-Pro-X-pSer/pThr/Glu/Asp^22,23^. These observations suggest that FBXW7 plays an essential role in diverse cellular processes depending on different cellular contexts in cancer cells.

In this study, we found that ERK3 is a binding partner of FBXW7 and that the interaction between the two proteins results in ERK3 ubiquitination and destabilization. Furthermore, this interaction was found to involve binding between the C34 domain (C34D) of ERK3 and the WD40 domain of FBXW7. In addition, mutations of Thr417 and Thr421 in ERK3 to alanine suppressed ERK3 ubiquitination, and ERK3 knockdown suppressed lung cancer cell proliferation. These observations suggest that the FBXW7-ERK3 degradation pathway plays an important role in cancer cell proliferation.

## Materials and methods

The general methodologies, including transfection, Western blotting, site-directed mutagenesis, real-time polymerase chain reaction, and focus formation assay, are described in detail in the Supplemental Materials. Material sharing can be requested by contacting the corresponding author (YY Cho, yongyeon@catholic.ac.kr).

### Reagents

Reagents for molecular and cellular biological studies, such as dimethyl sulfoxide (Cat #: D2650), cycloheximide (CHX; Cat #: 01810), and MG132 (Cat #: C2211), were purchased from Sigma-Aldrich (Sigma-Aldrich Korea, Gangnam, Seoul, Korea). TRIzol reagent (Cat #: 15596026) for RNA isolation was purchased from Thermo Fisher Scientific (Grand Island, NY, USA). Antibodies including anti-cyclin E (Cat #: sc-247), anti-Myc (Cat #: sc-40), anti-cullin 1 (Cat #: sc-17775), and anti-β-actin (Cat #: sc-47778) used for Western blotting and/or immunoprecipitation (IP) were obtained from Santa Cruz Biotechnology (Dallas, Texas, USA). An anti-FBXW7 antibody (Cat #: A301-721A) was obtained from Bethyl Laboratories, Inc. (Montgomery, Texas, USA). An anti-ERK3 antibody (Cat #: 4047) and an anti-HA-HRP antibody (Cat #: M180-7) were obtained from Cell Signaling Technology (Koram Biotech Corp., Gangnam, Seoul) and MBL International (Woburn, MA, USA), respectively. Protein G Sepharose beads (Cat #: 17-0618-02), which were used to pull down antibodies, were purchased from GE Healthcare (Chicago, IL, USA). A CellTiter 96^®^ AQueous One Solution Cell Proliferation Assay (Cat #: G3580) and crystal violet (Cat #: C1035) were purchased from Promega (Woods Hollow Road, Fitchburg, USA) and BIOSESANG (Sungnam, Gyeonggi, Korea), respectively.

### Cell culture

HEK293T cells, HCT116^*FBXW7+/+*^ cells, and HCT116^*FBXW7−/−*^ cells were cultured in Dulbecco’s modified Eagle’s medium (DMEM, Cat #: 10-013-CV, Corning Korea, Seoul, Korea) supplemented with 10% fetal bovine serum (FBS, Cat #: 35-015-CV, Corning Korea) containing 100 U penicillin and 100 µg/mL streptomycin (Cat #: 15070063, Thermo Fisher Scientific, Grand Island, NY, USA). A549 and H1299 lung cancer cells were cultured in RPMI-1640 (Cat #: 10-040-CV, Corning Korea) supplemented with 10% FBS containing 10 mM glutamine (Cat #: 25030081 Thermo Fisher Scientific), 100 U penicillin, and 100 µg/mL streptomycin (Cat #: 15070063, Thermo Fisher Scientific). FBXW7^+/+^ and FBXW7^−/−^ HCT116 cells (HCT116^*FBXW7+/+*^ and HCT116^*FBXW7−/−*^ cells) were kindly donated by Dr. Bert Vogelstein at the Sidney Kimmel Comprehensive Cancer Center at Johns Hopkins University^24^. All cell lines were maintained at 37 °C in a 5% CO_2_ incubator.

### Expression vectors

Mammalian two-hybrid vectors, pACT (VP16 fusion) and pBIND (Gal4 fusion), were purchased from Promega, and Myc- and HA-fusion vectors were purchased from TAKARA Bio Inc. (Kusatsu, Shiga, Japan). A His/Xpress fusion vector was purchased from Thermo Fisher Scientific, and a Flag-tag expression vector was purchased from Addgene (Watertown, MA, USA). Fusion expression vectors were constructed by inserting DNA fragments into the multicloning sites of pACT, pBIND, pCMV-Myc, pCMV-Flag, or pCMV-HA as indicated. We used pCDH-CMV-MCS-EF1-puro (Addgene) to overexpress FBXW7, ERK3-wt, and ERK3-mtPDM2 (designated pCDH-FBXW7, ERK3-wt, and ERK3-mtPDM2, respectively). All expression vectors were confirmed by DNA sequencing before use.

### Cell proliferation assay

To measure cell proliferation, A549 (1.5 × 10^3^ cells/well) or H1299 (2.0 × 10^3^ cells/well) lung cancer cells were seeded into 96‐well plates in 100 μL of cell culture medium and incubated for 2 h at 37 °C in a 5% CO_2_ incubator. At baseline, the absorbance was measured at 492 and 690 nm (to account for background debris, fingerprints, and other nonspecific absorbances) using MTS (3‐(4,5‐dimethylthiazol‐2‐yl)‐5‐(3‐carboxymethoxyphenyl)‐2‐(4‐sulfophenyl)‐2H‐tetrazolium)‐based CellTiter 96 Aqueous One Solution according to the manufacturer’s instructions (Promega, Madison, WI). Briefly, 20 μL of MTS solution was added per well, and the cells were incubated for 1 h at 37 °C in a 5% CO_2_ incubator. The reaction was stopped by adding 25 μL of 10% sodium dodecyl sulfate (SDS) solution to each well, and absorbances were measured immediately at 492 and 690 nm using an xMark^™^ Microplate Absorbance Spectrophotometer (Bio-Rad Laboratories, Hercules, CA, USA). Cell proliferation was evaluated by comparing sample absorbances measured over 96 h at 24 h intervals.

### Mammalian-two hybrid assay

Promega Checkmate Mammalian Two-Hybrid System protocols were used for the mammalian two-hybrid (M2H) assays. In brief, HEK293T cells were maintained in 10% FBS–DMEM, seeded into 48-well plates (2 × 10^4^ cells/well), and incubated with 10% FBS–DMEM for 18 h before transfection. DNAs, pACT kinases (including ERK3 and pBIND-FBXW7), and pG5-luciferase were combined at a molar ratio of 1:1:1. The total amount of DNA did not exceed 100 ng per well. Transfection was performed using H4000 (Engreen Biosystem Ltd., Beiyuan, Beijing China) according to the manufacturer’s instructions. For luciferase assays, cells were disrupted by adding passive lysis buffer (Cat #: E1941, Promega) and incubating for 30 min at room temperature with gentle shaking. Luciferase activities were measured automatically using a Dual-Luciferase^®^ Reporter Assay (Cat #: E1910, Promega), and data were obtained using a Victor X3 plate reader (PerkinElmer Korea, Guro, Seoul). The relative luciferase activities were determined using pG5-luciferase basal controls and normalized against *Renilla* luciferase activity. The pBIND vector was included as an internal control.

### Gene knockdown and ectopic expression

HEK293T cells were used for packaging of lentiviral or retroviral expression vectors to produce viral particles. The viral particles were then used to infect various cell lines. In brief, media containing secreted viruses were collected twice at 24 and 48 h after transfection and then filtered with 0.45 μm acetate syringe filters. Various cell lines were infected with the viruses in the presence of 1 μg/ml polybrene (Sigma-Aldrich Korea) and cultured for 48 h. Cells were selected using puromycin (5 μg/ml) for 3 days.

### Immunoprecipitation

Protein–protein interactions were investigated by IP and Western blotting. Antibodies (2 µg) in 700 µl of cell lysis buffer were added to cell lysates (300–400 µg) and hybridized with gentle inversion overnight at 4 °C. The lysates were then added to 30 µl of a 50% slurry of protein G Sepharose beads and incubated for 2 h at 4 °C. The interacting proteins were obtained by washing the beads with wash buffer (20 mM Tris at pH 8.0, 100 mM NaCl, 1 mM EDTA, and 0.5% NP-40) three times. The proteins were then resolved by SDS-PAGE and visualized by Western blotting using indicated antibodies.

### De novo ubiquitination assay

To quantify ubiquitination in cell culture, we cotransfected HEK293T cells with HA-ubiquitin or His-ubiquitin and Myc-ERK3 with or without FBXW7 as indicated. Cell lysates (300–400 μg) were immunoprecipitated using specific antibodies, and ubiquitinated ERK3 was detected using HA-tag-specific antibodies. Simultaneously, immunoprecipitated ERK3 levels were detected using endogenous ERK3 or Tag-specific antibodies. Ubiquitinated ERK3 was quantified by measuring the intensities of whole smear bands, and relative ERK3 ubiquitination values were obtained by equalizing the intensities of immunoprecipitated endogenous ERK3 or Tag-fusion ERK3.

### In vitro ubiquitination assay

The substrate proteins ERK3-wt and ERK3-mtPDM2 were prepared via coexpression of HA-GSK3β and Myc-Ab-conjugated bead pulldown in HCT116^*FBXW7−/−*^ cells. The beads were washed five times in NETN buffer (20 mM Tris-HCl [pH 8.0], 100 mM NaCl, 1 mM EDTA, 0.5% NP-40) and incubated with EBC solution (50 mM Tris-HCl [pH 8.0], 120 mM NaCl, 0.5% NP-40) for hybridization with immune-purified Flag-FBXW7. SCF^FBXW7^ complexes were prepared with an anti-Flag M2 affinity gel (Cat #:2220, Sigma-Aldrich Korea) and by elution with 3x Flag- peptide (Cat #: F4799, Sigma–Aldrich Korea) using the cell lysate from transiently Myc-cullin 1-, Myc-Rbx1-, and Flag-FBXW7-coexpressing HEK293T cells. For the in vitro ubiquitination assay, substrates (Myc-Ab-ERK3-wt and Myc-Ab-ERK3-mtPDM2) and SCF^FBXW7^ complexes were mixed together with the components of a ubiquitination kit (Cat #: BML-UW9920, ENZO Life Sciences, New York, NY, USA), and the assay was performed according to the manufacturer’s instructions. The ubiquitination of the ERK3-wt and ERK3-mtPDM2 proteins was visualized by Western blotting using an ERK3 antibody and an HRP-conjugated secondary antibody. The input was used to confirm the utilized protein amount for the in vitro ubiquitination assay by Western blotting.

### Cell synchronization and cell cycle release

A549 cells were used for cell cycle regulation assays, which involved HU-induced synchronization and subsequent release from the G_1_/S-phase using CDH-mock-, CDH-ERK3-wt-, and CDH-ERK3-mtPDM2-expressing cells. Briefly, the cells were treated with HU (2 mM) for 48 h, released by rinsing with 1× phosphate-buffered saline twice and refed with complete medium for the indicated times (referred to as “release times”). The cells were then trypsinized, fixed with ice-cold ethanol, and treated with RNase A (100 μg/mL) followed by propidium iodide (20 μg/mL). The cell cycle distributions were analyzed by flow cytometry (BD FACSCalibur^™^ flow cytometer, Franklin Lakes, NJ, USA).

## Results

### ERK3 is a novel binding partner of FBXW7

**Fig. 1:**
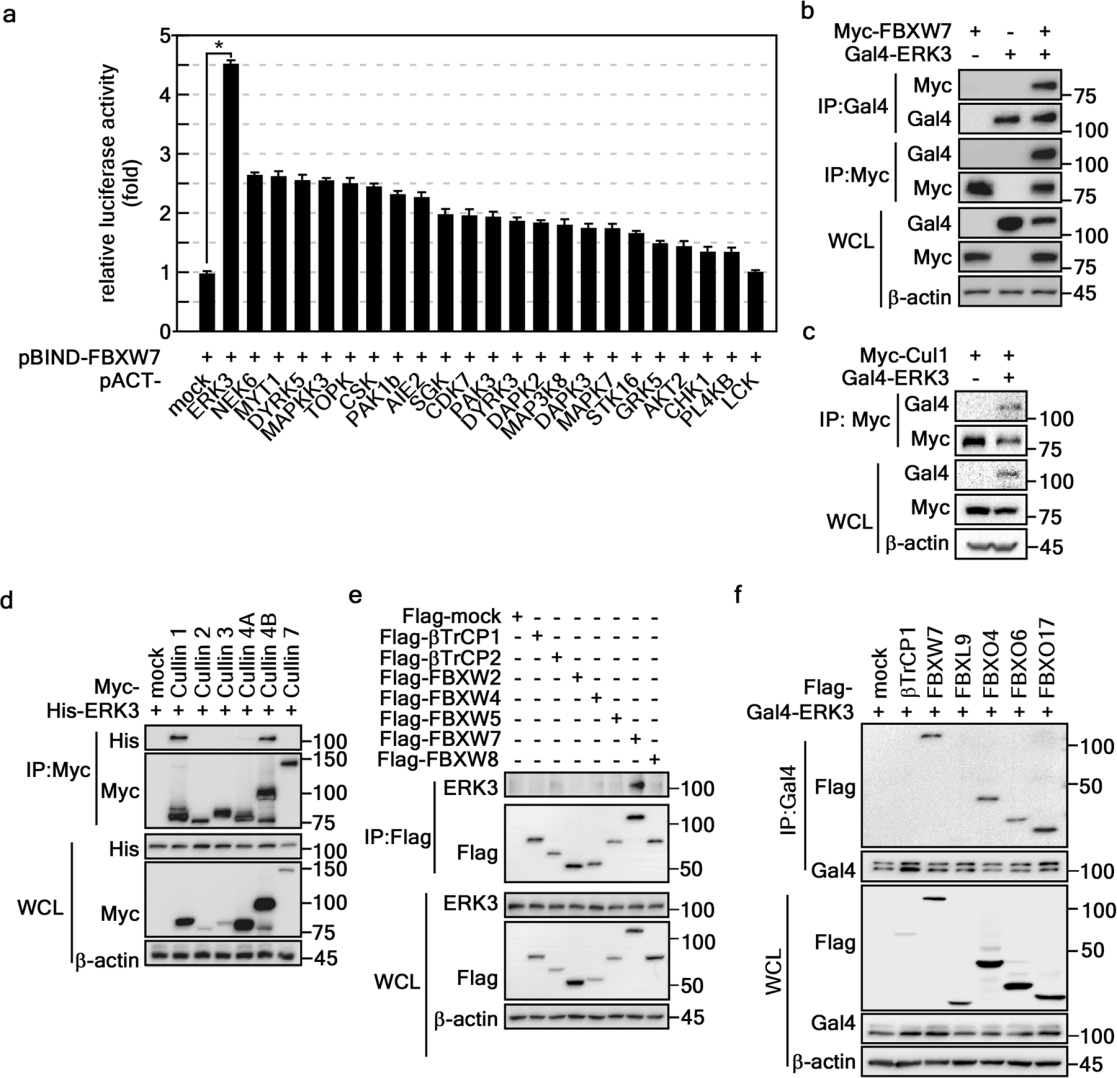
ERK3 is a novel binding partner of FBXW7. **a** Interaction screening of FBXW7 and kinases by mammalian two-hybrid assay. The interaction strengths between FBXW7 and each of the indicated kinases were assessed by measuring pG5-luciferase activity. The data are from a triplicate experiment and were normalized by Renilla luciferase activity. The values are shown ±SEMs. Significance; *p < 0.001 vs. pACT-mock control cells (Student’s *t* test). **b** Confirmation of the interaction between ERK3 and FBXW7. The interaction of FBXW7 and ERK3 was confirmed by IP (HEK293T cell lysate, 400 μg/lane). **c** ERK3 interacted with cullin 1. The participation of ERK3 in a cullin 1 complex was confirmed by IP (HEK293T cell lysate, 400 μg/lane). **d** Specificity of the ERK3 binding of cullin members. Specific ERK3 binding with cullin members was evaluated by IP. **e** ERK3 bound specifically to FBXW7 among FBXW subfamily members. Specific binding between ERK3 and FBXW subfamily members was evaluated by IP. **f** Specific ERK3 binding with FBXW7 and FBXO subfamily members. Specific ERK3 binding with FBXW7 and FBXO subfamily members was evaluated by IP. **b**–**f** β-Actin was used as the internal control to ensure equal protein loading. WCL, whole-cell lysate.

To identify the binding partner of FBXW7, Gal4-FBXW7 in the pBIND vector was used as a bait to screen for novel binding sites using MAPK kinases, which were recombined into the pACT expression vector. Mammalian two-hybrid assay results showed that FBXW7-ERK3 binding was approximately 4.5-fold greater than the binding between FBXW7 and negative control (vs. pBIND-FBXW7/pACT-mock) (Fig. 1a). The interaction between ERK3 and FBXW7 was confirmed by IP using HEK293T cell lysates ectopically expressing Myc-FBXW7 and Gal4-ERK3 (Fig. 1b). Since FBXW7 is a member of the cullin 1 SCF complex family^25^, we first confirmed the binding of ERK3 to cullin 1 by IP. The results demonstrated that ERK3 is bound to cullin 1 (Fig. 1c). We further evaluated specific binding between ERK3 and isotype(s) of cullin family members (i.e., cullin 1, 2, 3, 4A, 4B, and 7) and observed that ERK3 coimmunoprecipitated with cullin 1 and 4B (Fig. 1d). Specific endogenous ERK3 binding with FBXW subfamily members, including βTrCP1, βTrCP2, FBXW2, FBXW4, FBXW5, FBXW7, and FBXW8, was evaluated using ex vivo Flag-tag pulldown assays. We found that immunoprecipitated ERK3 was detected only in the presence of FBXW7 (Fig. 1e). Using expression constructs, we further found that ERK3 interacted with FBXW7 and FBXO 4, 6, and 17 (Fig. 1f). These results suggest that ERK3 may also interact with other F-box subfamily members. Since our laboratory possessed many constructs that allowed a detailed investigation of the molecular mechanisms of FBXW7^25^, we focused on the interaction between FBXW7 and ERK3. Our results showed that the ERK3/FBXW7 interaction is specific for the FBXW subfamily of F-box proteins.

### FBXW7 destabilizes ERK3 by ubiquitination

**Fig. 2:**
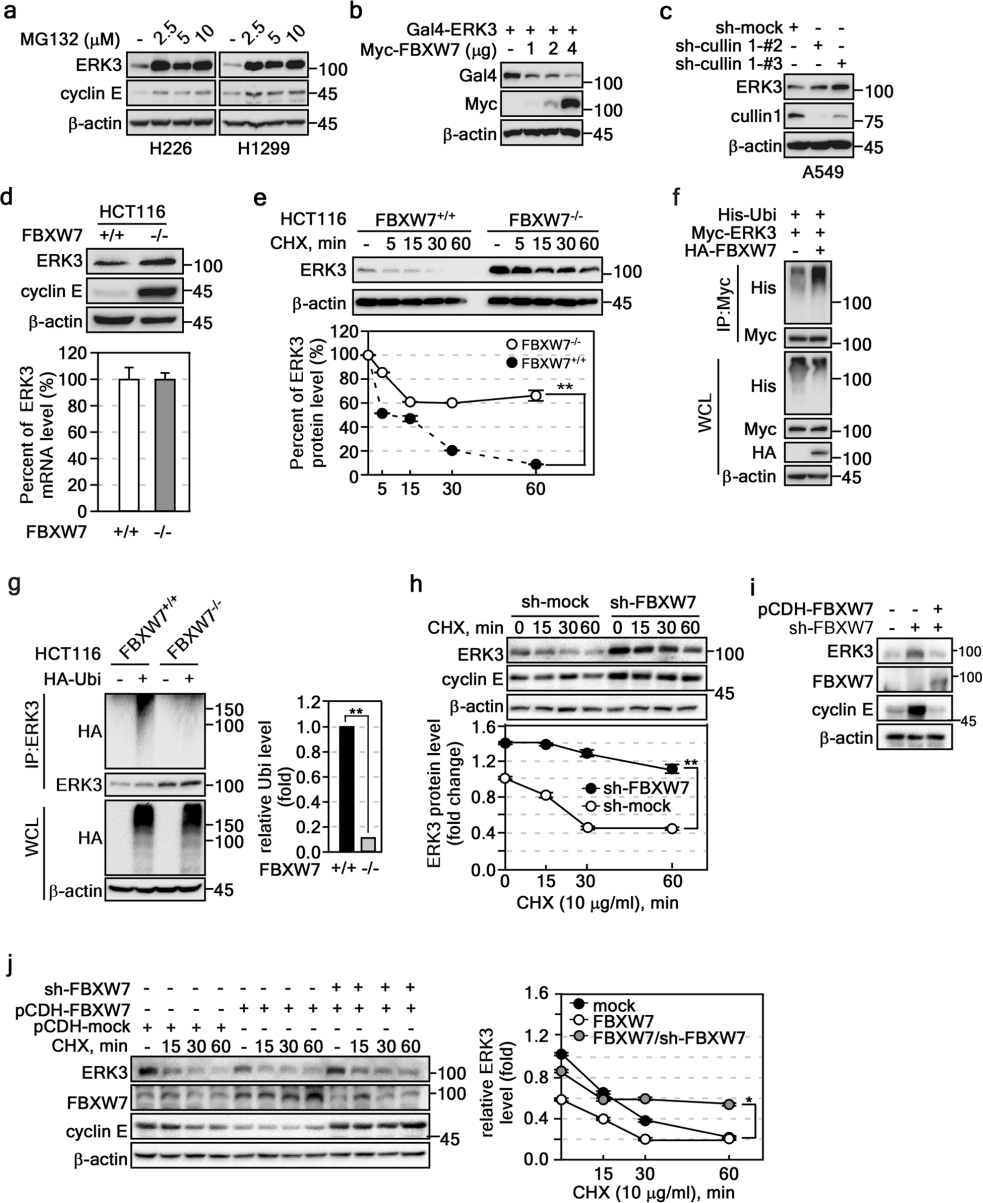
FBXW7 induced the ubiquitination of ERK3. **a** MG132 restored ERK3 protein levels. Lung cancer cells (H226 and H1299 cells) were treated with MG132 (10 μM) for 4 h, and cell lysates (30 μg) were subjected to Western blotting to assess ERK3 protein levels. **b** FBXW7 downregulated ERK3 protein levels. FBXW7-mediated ERK3 protein level changes were assessed by Western blotting (30 μg protein/lane). **c** Cullin 1 knockdown increased ERK3 protein levels. ERK3 protein levels in cullin-knockdown cells (30 μg protein/lane) were assessed by Western blotting. **d** *Upper panels*, FBXW7 deficiency increased ERK3 protein levels. ERK3 protein levels in cell lysates (30 μg) of HCT116^*FBXW7+/+*^ and HCT116^*FBXW7−/−*^ cells were determined by Western blotting. Cyclin E: positive control. *Graphs*, ERK3 gene expression was not involved in ERK3 destabilization. ERK3 mRNA levels in HCT116^*FBXW7+/+*^ or HCT116^*FBXW7−/−*^ cells were evaluated by real-time PCR. **e** FBXW7 attenuated ERK3 degradation. *Upper panels*, ERK3 protein levels in HCT116^*FBXW7+/+*^ and HCT116^*FBXW7−/−*^ cells were determined by Western blotting (30 μg cell lysate/lane, CHX; 10 μg/ml). *Graphs*, The ERK3 band intensities were normalized to the β-actin intensity. **f** FBXW7 increased ERK3 ubiquitination. FBXW7-mediated ERK3 ubiquitination was evaluated by IP (400 μg cell lysate)/Western blotting. **g** Depletion of FBXW7 suppressed ERK3 ubiquitination. *Left panels*, The endogenous ERK3 ubiquitination content in HCT116^*FBXW7+/+*^ and HCT116^*FBXW7−/−*^ cells was evaluated by IP (500 μg cell lysate/lane)/Western blotting. *Graphs*, The intensities of HA-Ubi ERK3 bands were measured and normalized using ImageJ (ver. 1.52a). **h** *Upper panels*, FBXW7 knockdown attenuated ERK3 degradation. The ERK3 turnover rate in FBXW7-knockdown A549 cells was evaluated by Western blotting (20 μg cell lysate/lane, CHX; 10 μg/ml). The ERK3 band intensities were normalized to the β-actin intensity. Cyclin E, positive control. **i** FBXW7 reintroduction into FBXW7-knockdown cells suppressed ERK3 protein levels. The ERK3 protein levels in A549 stable cells stably expressing sh-mock, sh-FBXW7, or sh-FBXW7/Lenti-pCDH-FBXW7 were determined by Western blotting (20 μg cell lysate/lane). **j** *Left panels*, FBXW7 knockdown decreased the ERK3 turnover rate, which was increased by FBXW7 overexpression. The ERK3 turnover rate in A549 cells stably expressing Lenti-pCDH-FBXW7 or Lenti-pCDH-FBXW7/sh-FBXW7 was determined by Western blotting (20 μg cell lysate/lane, CHX; 10 μg/ml). *Graphs*, The ERK3 band intensities were normalized to the β-actin intensity. **a-j** β-Actin was used as the internal control to ensure equal protein loading. **a-j** The data are from three independent experiments. **d**, **e**, **g**, **h**, **j** The values are shown ± the SEMs. Significance; *p < 0.05, **p < 0.01 vs. indicated control by Student’s *t* test. **f**, **g** WCL, whole-cell lysate.

After finding that ERK3 was a novel binding partner of FBXW7 (Fig. 1a–c), we examined whether ERK3 protein content was regulated via proteasomal degradation. To examine this hypothesis, we treated H226 and H1299 lung cancer cells with MG132, a proteasomal degradation inhibitor. The results demonstrated that ERK3 protein levels were markedly higher in MG132-treated cells than in nontreated controls (Fig. 2a). As we have reported previously^25^, MG132 treatment restored cyclin E protein levels, indicating that ERK3 protein levels were regulated by the proteasomal degradation pathway (Fig. 2a). Notably, increasing the ectopic expression of FBXW7 dose-dependently reduced ERK3 protein levels (Fig. 2b). Moreover, cullin 1 knockdown in A549 lung cancer cells elevated ERK3 protein levels (Fig. 2c). When cullin 1-knockdown A549 lung cancer cells were treated with cycloheximide (CHX), ERK3 protein levels were sustained. On the other hand, sh-mock A549 cells showed gradual, time-dependent reductions in ERK3 protein levels after CHX treatment (Supplementary Fig. 1a), which indicated that FBXW7 regulates ERK3 protein levels via a proteasomal degradation pathway. This result was further supported by higher ERK3 protein levels in HCT116^*FBXW7−/−*^ cells than in HCT116^*FBXW7+/+*^ cells (Fig. 2d, upper panels). Since ERK3 mRNA levels were similar in these cells (Fig. 2d, bottom graph), we concluded that the elevated ERK3 protein levels were caused by enhanced protein stability and not by gene expression. Notably, cycloheximide (CHX) treatment demonstrated that the degradation rate of ERK3 proteins in HCT116^*FBXW7−/−*^ cells was slower than that in HCT116^*FBXW7+/+*^ cells (Fig. 2e). As expected, coexpression of ERK3 and FBXW7 dramatically increased ERK3 ubiquitination compared to that achieved with ERK3 alone (Fig. 2f). Furthermore, ERK3 ubiquitination was markedly abolished in HCT116^*FBXW7−/−*^ cells (Fig. 2g). In contrast to FBXW7 overexpression, FBXW7 knockdown in A549 cells (Supplementary Fig. 1b) increased not only the total protein level of ERK3 but also ERK3 turnover after CHX treatment (Fig. 2h). The increased levels of cyclin E induced by sh-FBXW7 provided evidence that the experiment was suitable (Fig. 2h). Notably, the reintroduction of FBXW7 overexpression plasmids into FBXW7-knockdown cells resulted in dramatic reductions in ERK3 levels (Fig. 2i). In contrast, the reduced ERK3 protein stability caused by FBXW7 overexpression compared to that in the mock control group was attenuated by knockdown of FBXW7 in a time-dependent manner (Fig. 2j). However, FBXW7 knockdown resulted in a gradual degradation trend, indicating the presence of other ERK3 ubiquitination enzymes. Although Figs. 1d, f and 2j suggest that other F-box proteins may be involved in the regulation of ERK3, the results overall indicate that FBXW7-mediated ERK3 ubiquitination regulates ERK3 protein stability via the proteasomal degradation pathway.

### The C34D of ERK3 interacts with the WD40 domain of FBXW7

**Fig. 3:**
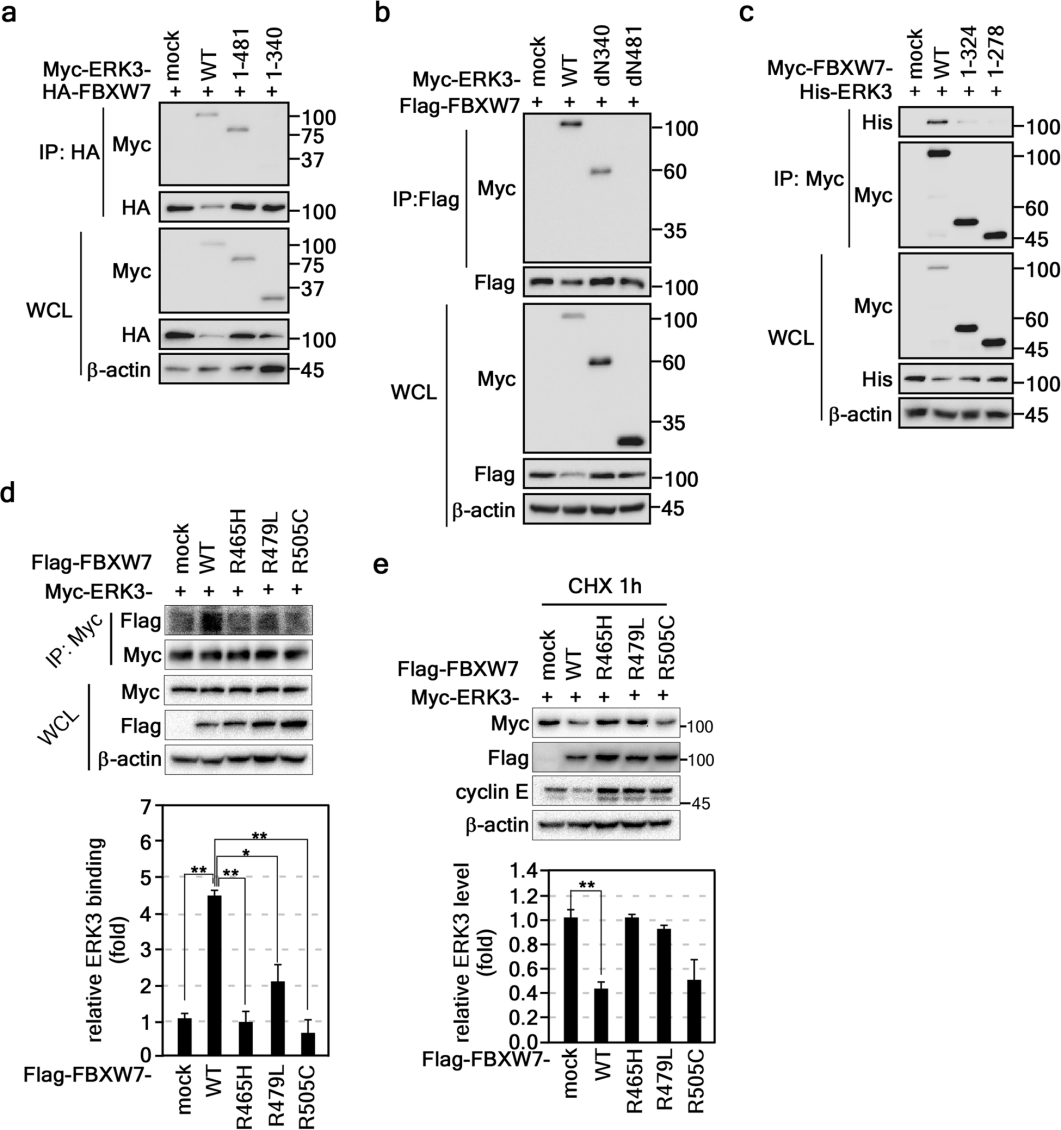
The C34D of ERK3 interacted with the WD40 domain of FBXW7. **a** Identification of ERK3 domains that interact with FBXW7. The ERK3 domains interacting with FBXW7 were determined by IP (HEK293T cell lysate, 300 μg cell lysate/lane) and Western blotting. **b** Involvement of the ERK3 C34D in its interaction with FBXW7. The ERK3 C34D was determined to interact with FBXW7 by IP (HEK293T cell lysate, 300 μg)/Western blotting. **c** Determination of FBXW7 domains that interact with ERK3. The FBXW7 interaction domains interacting with ERK3 were determined by IP (HEK293T cell lysate, 300 μg) and Western blotting. **d** *Upper panels*, Confirmation that the WD40 domain interacts with ERK3. The interactions of FBXW7 proteins containing each mutation in the WD40 domain and ERK3 were determined by IP and Western blotting (MG132; 10 μM, 4 h). *Graphs*, Immunoprecipitated FBXW7 protein levels were normalized to the levels of immunoprecipitated Myc-ERK3. **e** *Upper panels*, ERK3 protein levels were sustained in FBXW7 mutants. The ERK3 protein levels in HEK293T cells transiently expressing ERK3 and each FBXW7 mutation as indicated were determined by Western blotting (CHX; 10 μg/ml, 1 h). *Graphs*, The Myc-ERK3 band intensities were normalized to the β-actin intensity. **a**–**e** β-Actin was used as the internal control to ensure equal protein loading. WCL, whole-cell lysate.

To identify the domain of ERK3 responsible for its interaction with FBXW7, we constructed serial deletion mutants of ERK3 harboring amino acids 1–721 (Myc-ERK3-1–721), 1–481 (Myc-ERK3-1-481), or 1–340 (Myc-ERK3-1-340) (Supplementary Fig. 2a). IP experiments using HEK293T cell lysates coexpressing HA-FBXW7 and Myc-ERK3-1–721, Myc-ERK3-1–481, or Myc-ERK3-1–340 showed that FBXW7 and ERK3 formed a complex in Myc-ERK3-1–721 and Myc-ERK3-1–481 (Fig. 3a), suggesting that FBXW7 interacts with the C34D of ERK. IP using Flag-FBXW7 and ERK3 mutants with serial deletion from the N-terminus (Myc-ERK3-dN340) and amino acids 481–721 (Myc-ERK3-dN481) (Supplementary Fig. 2b) showed that the C-terminal domain of ERK3 did not interact with FBXW7 (Fig. 3b). On the other hand, when we used FBXW7 serial deletion mutants (Supplementary Fig. 2c), we observed that ERK3 interacted with full-length FBXW7 (Myc-FBXW7-1–707) but not with the WD40 domain-deleted mutant (Myc-FBXW7-1–324) or the WD40/F-box domain-deleted mutant (Myc-FBXW7-1–278) (Fig. 3c). After determining the domain in ERK3 responsible for the interaction with FBXW7, it was necessary to determine the binding domain(s) of FBXW7. Based on the literature indicating that Arg465, Arg479, and Arg505 of FBXW7 play a critical role in the interaction of FBXW7 substrate^25^, we investigated the interactions of FBXW7 mutants harboring R465H, R479L, or R505C with ERK3. The IP results indicated that FBXW7 mutation dramatically reduced the interaction with ERK3 (Fig. 3d). Notably, when the ERK3 was coexpressed with each of FBXW7-wt or -mutants, the decreased ERK3 protein levels were observed in FBXW7-wt, but not in FBXW7-mutants (Fig. 3e). Cyclin E protein levels also showed patterns similar to those of ERK3 levels, indicating that ERK3 protein levels were regulated by interaction with the WD40 domain of FBXW7 (Fig. 3e). These results indicate that the C34D of ERK3 interacts with the WD40 domain of FBXW7.

### Thr417 and Thr421 of ERK3 act as degron motifs

**Fig. 4:**
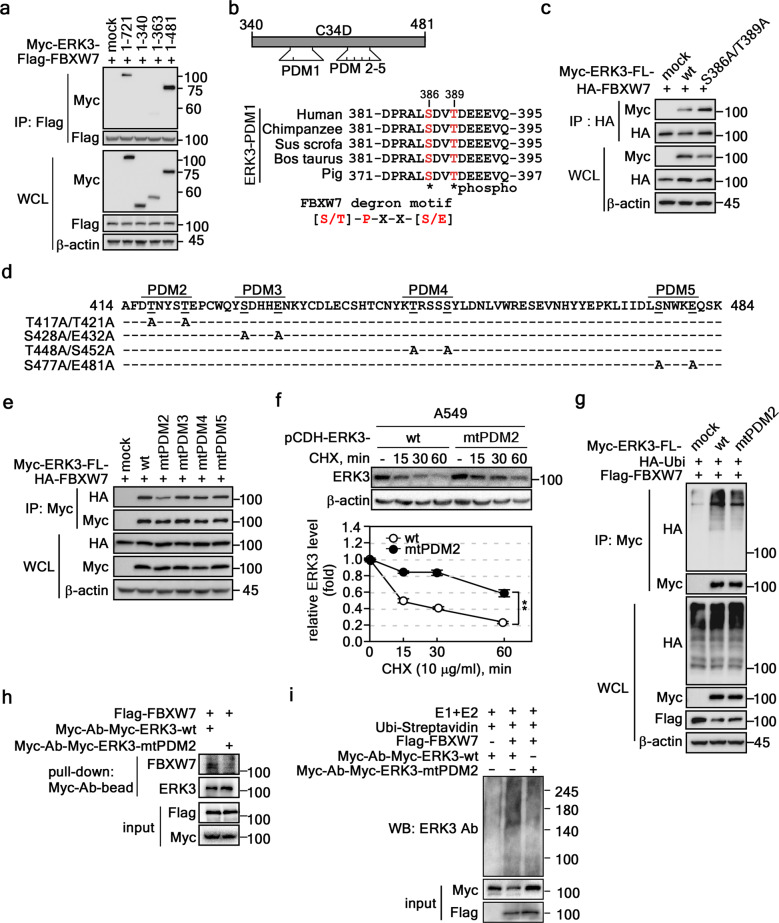
Thr417/Thr421 of ERK3 acts as a degron motif to bind FBXW7. **a** The ERK3 C34D-364–481 region interacted with FBXW7. The interaction of amino acids 364–481 of the ERK3 C34D with FBXW7 was determined by IP (HEK293T cell lysate, 300 μg/lane)/Western blotting. **b** *Upper panels*, Vector maps indicating the locations of putative degron motifs (PDMs) for FBXW7 in the ERK3 C34D. *Bottom panels*, Amino acid alignment of PDM1. **c** Ser386/Thr389 did not serve as an ERK3 degron for FBXW7. Ser386/Thr389 involvement in ERK3-FBXW7 binding was determined by IP (HEK293T cell lysate, 300 μg/lane)/Western blotting. **d** Amino acid alignments and the locations of PDM2-5 of ERK3 in the C34D. **e** Interaction between FBXW7 and ERK3-mtPDM2-5 mutants. The interaction of FBXW7 and each Myc-ERK3-FL-mtPDM was evaluated by IP (HEK293T cell lysate, 300 μg)/Western blotting. **f** *Upper panels*, ERK3-mtPDM2 attenuated protein degradation. The turnover of ERK3 in A549 cells stably expressing ERK3-wt and ERK3-mtPDM2 was determined by Western blotting (CHX; 10 μg/ml). *Graphs*, The ERK3 band intensities were normalized to the β-actin intensity. The data were obtained from three independent experiments. The values are shown with ±SEMs. Significance; **p < 0.05 vs. wt control by Student’s *t* test. **g** Myc-ERK3-FL-mtPDM2 reduced FBXW7-mediated ubiquitination. FBXW7-mediated ERK3-FL and ERK3-FL-mtPDM2 ubiquitination was compared by IP (HEK293T cell lysate, 300 μg/lane)/Western blotting. **h** ERK3-mtPDM2 decreased complex formation with FBXW7 ex vivo. Immunopurified Flag-FBXW7 proteins were subjected to complex formation assays with Myc-Ab-myc-ERK3-WT or Myc-Ab-myc-ERK3-mtPDM2 beads. The beads were washed, and ERK3 binding was determined by Western blotting. The input was used to compare the protein amounts for these reactions. **i** FBXW7 ubiquitinated ERK3 in vitro. The proteins prepared in (**h**) were subjected to confirmation of the ERK3 ubiquitination degree using an in vitro ubiquitination kit as described in the Materials and Methods. Ubiquitinated ERK3 protein levels were determined by Western blotting. **a**, **c**, **e**–**g** β-Actin was used as an internal control to ensure equal protein loading. WCL, whole-cell lysate.

To investigate the molecular mechanisms responsible for the interaction between the C34D of ERK3 and the WD40 domain of FBXW7, we first tried to narrow down the region responsible for ERK3 binding. IP using constructs with deletion of the C34D of ERK3 (Supplementary Fig. 3a) showed that Myc-ERK3-1–721 and Myc-ERK3-1–481 immunoprecipitated with FBXW7, whereas Myc-ERK3-1–363 and Myc-ERK3-1–340 did not (Fig. 4a). These results support the notion that the degron motif(s) of ERK3 spanning amino acids 364–481 are recognized by the WD40 domain of FBXW7. Accordingly, we analyzed the sequence of ERK3 at amino acids 364–481 and found 5 putative degron motifs (PDMs), which were designated PDMs 1–5 (Fig. 4b, *maps*). We first constructed a mutated expression vector of full-length ERK3 harboring a mutation of Ser386/Thr389 to alanine at PDM1 (Myc-ERK3-FL-mtPDM1) (Fig. 4b, *bottom sequence alignment*). IP using cell lysates transiently expressing HA-FBXW7, HA-FBXW7/Myc-ERK3-FL-wt, or HA-FBXW7/Myc-ERK3-FL-mtPDM1 showed that this PDM1 mutation did not alter the interaction between FBXW7 and ERK3 (Fig. 4c). These results indicate that PDM1 is not a degron motif of ERK3 for FBXW7. Next, we constructed four independent expression vectors harboring a double mutation from PDM2 to PDM5. These vectors were designated Myc-ERK3-FL-T417A/T421A, Myc-ERK3-FL-mtPDM2; Myc-ERK3-FL-S428A/E432A, Myc-ERK3-FL-mtPDM3; Myc-ERK3-FL-T448A/S452A, Myc-ERK3-FL-mtPDM4; and Myc-ERK3-FL-S477A/E481A, Myc-ERK3-FL-mtPDM5 (Fig. 4d). IP results obtained using HA-FBXW7 and these expression vectors showed that mutation of ERK3 Thr417 and Thr421 to alanine (Myc-ERK3-FL-mtPDM2) dramatically reduced its interaction with FBXW7 (Fig. 4e), whereas Myc-ERK3-FL-mtPDM3, Myc-ERK3-FL-mtPDM4, and Myc-ERK3-FL-mtPDM5 had little effect (Fig. 4e). Importantly, the reduction rate of the Myc-ERK3-FL-mtPDM2 protein was lower after CHX treatment than the reduction rate for the ERK3-wt (Fig. 4f and Supplementary Fig. 3b). To determine whether the lower reduction rate of Myc-ERK3-FL-mtPDM2 protein was due to the suppression of ubiquitination, we conducted an FBXW7-mediated ERK3 ubiquitination experiment. As expected, the ERK3 ubiquitination content was markedly lower for Myc-ERK3-FL-mtPDM2 than for ERK3-wt (Fig. 4g). Notably, ex vivo binding of FBXW7 and Myc-ERK3-wt or Myc-ERK3-mtPDM2 demonstrated that mutation of ERK3 at PDM2 suppressed the interaction with FBXW7 (Fig. 4h). Furthermore, an in vitro ubiquitination experiment demonstrated that mutations at PDM2 of ERK3 prevented FBXW7-mediated ERK3 ubiquitination (Fig. 4i). These results demonstrated that the ubiquitination reduction in Myc-ERK3-mtPDM2 was due to disruption of the ERK3-FBXW7 interaction. These results show that FBXW7 modulates ERK3 stability by interacting with Thr417 and Thr421 in the C34D of ERK3 using its WD40 domain via a ubiquitination-mediated proteasomal degradation pathway.

### The FBXW7-ERK3 signaling axis regulated the proliferation of lung cancer cells

**Fig. 5:**
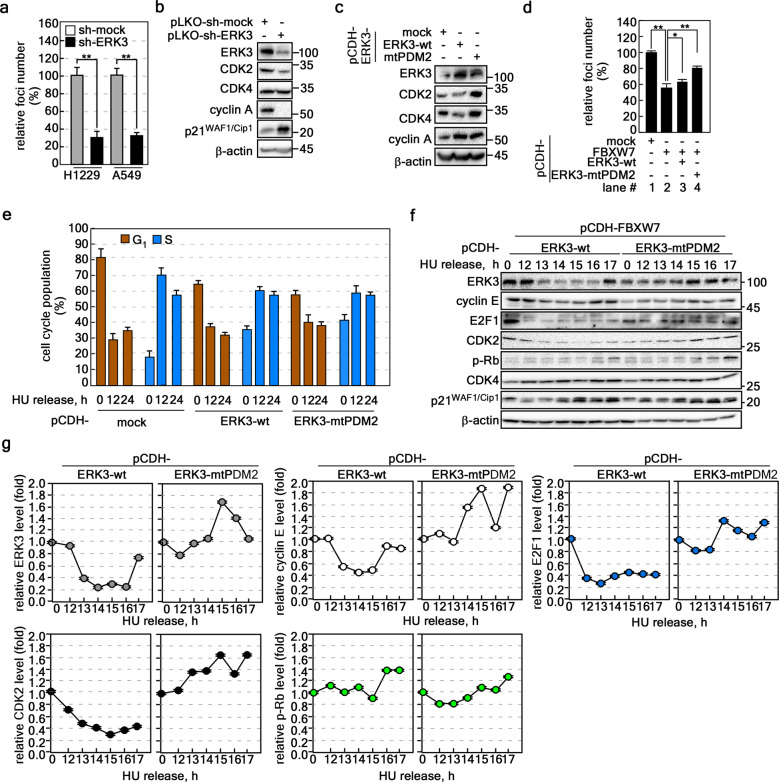
S-phase checkpoint regulation by the FBXW7-ERK3 axis regulates focus formation in cancer cells. **a** ERK3 knockdown inhibited the proliferation of lung cancer cells. The effects of ERK3 knockdown on the proliferation of H1299 and A549 cells were evaluated by a focus formation assay. **b** ERK3 knockdown reduced the protein levels of G_1_/S-phase cell cycle regulators. The levels of cell cycle regulation-related proteins were evaluated by Western blotting (ERK3-knockdown A549 cell lysate, 30 μg/lane). **c** Ectopic expression of ERK3 increased the protein levels of G_1_/S-phase cell cycle regulators. The protein level changes of G_1_/S-phase cell cycle regulators caused by ectopic expression of ERK3-wt or ERK3-mtPDM2 were evaluated by Western blotting (A549 cell lysate, 30 μg/lane). **d** Ectopic expression of ERK3-mtPDM2 reversed the FBXW7-mediated inhibition of focus formation. A549 cells stably expressing mock, ERK3-wt, FBXW7, or ERK3-mtPDM2 were used to investigate the role of the FBXW7-ERK3 axis in focus formation. **e-g** ERK3 induced the G_1_/S-phase cell cycle transition. The A549 cells in **c** were synchronized at the G_1_ phase of the cell cycle by HU treatment (48 h) and then released via culture in a complete growth medium. At each time point after HU release, flow cytometry was used to analyze the cell cycle distribution (**e**), and Western blotting was used to confirm the protein oscillations (**f**). The graphs in **g** show the oscillation pattern of each indicated protein after HU release. The band intensities of each indicated protein were normalized to the β-actin intensity. **a**, **d** The data were obtained from three independent experiments. The values are shown ±SEMs. Significance; *p < 0.05, **p < 0.01 vs. nontreated control or sh-mock by Student’s *t* test. **b**, **c**, **f** β-Actin was used as an internal control to ensure equal protein loading.

To examine the roles of ERK3-mediated cancer cell proliferation, we knocked down ERK3 in H1299 and A549 lung cancer cells (Supplementary Fig. 4a). Using these cells, we confirmed that although the inhibitory rates were different for each ERK3-knockdown construct, the inhibitory patterns showed that ERK3 knockdown severely reduced cancer cell proliferation (Supplementary Fig. 4b). Notably, ERK3 knockdown resulted in the formation of substantially fewer foci in A549 cells than sh-mock treatment in control cells (Supplementary Fig. 4c). Since sh-ERK3-#3 showed average inhibition of cell proliferation (Supplementary Fig. 4b), we selected sh-ERK3-#3 for further analysis of ERK3’s role in cancer cell proliferation. In addition, knockdown of ERK3 in both cell lines induced cell growth arrest and significantly reduced focus formation (Fig. 5a, Supplementary Fig. 4d). Interestingly, we found that ERK3 knockdown decreased CDK2 and cyclin A protein levels and increased p21^WAF1/Cip1^ protein levels (Fig. 5b), indicating that the ERK3 protein might play a role in cell cycle regulation. Notably, ERK3-mtPDM2 overexpression increased the CDK2, CDK4, and cyclin A protein levels (Fig. 5c). These results indicated that ERK3 might be involved in the induction of cell proliferation. Since ectopic expression of FBXW7 has been shown to suppress cell proliferation^25^, ectopic expression of ERK3 or ERK3-mtPDM2 might restore FBXW7-mediated inhibition of cell proliferation. As expected, overexpression of FBXW7 decreased focus formation (Fig. 5d, Lane 1 vs. 2). The reduction in focus formation was somewhat attenuated by overexpression of ERK3-wt or ERK3-mtPDM2 (Fig. 5d, Lane 2 vs. 3 or 4). The restoration ratio of focus formation was higher in ERK3-mtPDM2-overexpressing cells than in ERK3-wt cells (Fig. 5d, Lane 3 vs. 4). However, the restored focus formation did not exceed the levels of the mock control cells (Fig. 5d, Lanes 1 vs. 3 and 4). These results indicated that ERK3 might play a positive role in cell proliferation and focus formation. To clarify the role of ERK3 in cell proliferation, we analyzed the effect of ERK3 on cell cycle distribution by using hydroxyurea (HU) to synchronize cells at the G_1_ phase of the cell cycle and releasing ERK3 from G_1_ arrest by moving the cells to HU-free complete medium to initiate DNA replication. Flow cytometry indicated that mock-expressing stable cells showed an ~52% reduction in the G_1_-phase cell cycle population and an ~51% increase in the S-phase cell cycle population at the 12 h time point (Fig. 5e). We observed that the ectopic expression of ERK3-wt suppressed the G_1_-phase cell cycle population by approximately 26% and increased the S-phase cell population by approximately 24% at the 12 h time point (Fig. 5e). Compared to ERK3-wt overexpression, ERK3-mtPDM2 overexpression also resulted in a similar increase and decrease in the cell cycle populations at the 12 h time point (Fig. 5e). Importantly, the HU-induced 80% G_1_ accumulation in mock-overexpressing cells was reduced to approximately 60–65% by ERK3-wt or ERK3-mtPDM2 overexpression (Fig. 5e). In contrast to the G_1_ population, the HU-treated 18% S-phase cell cycle population in mock-overexpressing cells was reduced to approximately 38–40% by ERK3-wt or ERK3-mtPDM2 overexpression (Fig. 5e). These results suggested that ERK3 acted as a positive regulator to enhance the rapid progression of the cell cycle from G_1_ phase to the S phase. This hypothesis was supported by monitoring the protein levels of cell cycle regulators, including the CDK2, CDK4, cyclin E, E2F1, and phospho-retinoblastoma (Rb) proteins (Fig. 5f, g). The results indicated that ERK3-wt protein levels decreased gradually by 16 h after HU release and were increased at 17 h after HU release (Fig. 5f, *top panel*, and **g** *left-top*). Interestingly, ERK3-mtPDM2 protein levels were sustained and even increased in a time course after HU release (Fig. 5f, *top panel*, and **g** *left-top*). Importantly, although CDK4 and p21^WAF1/Cip1^ protein levels were not significantly changed (Fig. 5f, 6th–7th *panels from top*), cyclin E, CDK2, E2F1, and phospho-Rb protein levels were increased in ERK3-mtPDM2-overexpressing A549 cells in a time course after HU release compared to the levels in ERK3-wt-overexpressing A549 cells (Fig. 5f, g). Taken together, these results indicate that ERK3 plays an important role in the G_1_/S-transition cell cycle checkpoint.

## Discussion

In the present study, our data showed that FBXW7 overexpression inhibited focus formation in A549 cells more than ERK3 overexpression (Fig. 5d). Interestingly, ERK3 and FBXW7 coexpression slightly restored focus formation, which was suppressed by FBXW7 alone (Fig. 5d). The restoration rate of focus formation was significantly increased by coexpression of ERK3-mtPDM2 and FBXW7 (Fig. 5d). These results suggest that ERK3 has the potential to enhance cell proliferation and focus formation. However, the expression of ERK3-wt or ERK3-mtPDM2 was not fully restored or increased compared to that in mock-expressing control cells (Fig. 5d). However, we did not observe any cell death due to ERK3 overexpression. These results suggest that ERK3 might play an important role when cells are situated in some conditions that are not yet known. On the other hand, knockdown of ERK3 severely suppressed H1299 and A549 cell proliferation and eventually caused proliferation arrest (Supplementary Fig. 4b–d). These results also suggest some specific role of ERK3 in the determination of cell fate under particular conditions. This idea is supported by the discovery that the activating phosphorylation of ERK3/ERK4 is not modulated by classic MAPK stimuli or by any other extracellular stimuli examined^26^. Interestingly, ERK3 is present in some snRNP-containing structural constituents, especially in perichromatin fibrils, where transcriptional and cotranscriptional splicing of mRNAs occur^27^. Moreover, the interaction of ERK with cyclin D3 and MK5 suggests that ERK3’s function is linked to cell cycle progression and differentiation^28^. However, the upstream stimuli and kinases directing ERK3 activation and subcellular localization have not been clearly elucidated thus far.

ERK3-deficient C57BL/6 mice are nonviable and show pulmonary hypoplasia and incomplete type II pneumocyte differentiation^29^. However, these observations were recently re-evaluated in two publications that confirmed that the observed phenotype is likely attributable to off-target effects^29–31^. Our results confirm that ERK3 protein levels are positively correlated with the G_1_/S cell cycle transition in A549 lung cancer cells (Fig. 5e). Although the nuclear substrates of ERK3 that trigger cellular responses are unknown, ERK3 is required for the nuclear abundance of c-Jun protein and the DNA-binding activity of AP-1, which consequently regulates IL-8 and chemotaxis^16^. These results suggest that ERK3 may also be involved in cell proliferation. Since MK5 is a well-known direct downstream kinase that is targeted by ERK3 and since ERK3 activation upregulates MK5^32^, the phenotypes manifested by ERK3 or MK5 loss-of-function might be similar. For example, the L290V substitution in ERK3 has been observed in lung and skin cancer samples, and ERK3 mRNA levels in oral cancer tissues were 5- to 8-fold higher than those in healthy tissues in 90% of patients (*n* = 37/41)^33,34^. In another study, peripheral blood cell analysis showed that ERK3 mRNA levels were elevated in ~61% (*n* = 8/13) of oral squamous cell carcinoma patients but undetectable or low in healthy individuals 74%, (*n* = 23/31)^35^. These results indicate that ERK3 might be involved in cancer development.

To investigate the mechanism of ERK3-mediated cancer cell proliferation, we used H1299 and A549 lung cancer cells. Since ERK3 is classified as an atypical MAP kinase ubiquitously expressed in human tissues and since H1299 and A549 cells carry mutations in the same family of Ras proteins (N-Ras and K-Ras, respectively), we hypothesized that these two cells may exhibit similar responses to ERK3 protein levels or activity. Surprisingly, different effects of knockdown of the ERK3 gene were observed (Supplementary Fig. 4a, b). Thus, to avoid biases caused by the knockdown effect of the single clone sh-RNA, several different clones of sh-RNA were generally examined to determine whether similar patterns of phenotypes were observable. If the effects of gene knockdown are shown to have similar patterns in several different clones, the clones showing these results can be selected, as indicated by our current results (Fig. 5a–g). Although our results showed different inhibition rates of cell proliferation depending on the clone, the similar patterns of cell proliferation inhibition caused by ERK3 knockdown indicated that ERK3 might positively regulate cell proliferation (Supplementary Fig. 4a, b). Moreover, we found that FBXW7 acts as an ERK3 stability regulator. Since FBXW7 acts as a tumor suppressor by targeting oncogenes for degradation^36^, ERK3 ubiquitination, and degradation mediated by FBXW7 support the oncogenic function of ERK3. However, a recent study reported that although BRAF increases ERK3 transcription and stability, ERK3 expression in melanoma cells suppresses cell growth^37^. Although the physiological role of the ERK3 protein remains controversial, our results consistently support the idea that FBXW7 acts as a tumor suppressor. Thus, we believe that FBXW7-mediated ERK3 degradation suppresses cancer cell proliferation. We initially thought that ERK3 might accelerate cell cycle progression in cancer cells. However, this view was contradicted by the results of the focus formation assays on cells stably expressing FBXW7, ERK3-wt, and/or ERK3-mtPDM2 and by the results of the HU pulse/chase experiments. Surprisingly, we found that FBXW7 alone suppressed focus formation in A549 cells, whereas ERK3-mtPDM2 restored the number of foci after FBXW-mediated suppression (Fig. 5d and Supplementary Fig. 4e). In addition, compared to the expression of FBXW7 alone, coexpression of FBXW7 and ERK3-wt did not significantly restore focus formation (Fig. 5d). These results indicate that although ERK3 seemingly plays an important role in cancer cell proliferation, the molecular mechanisms responsible for regulating ERK3 protein levels and activities are complex. Moreover, since ERK3 protein levels were gradually decreased in HCT116^*FBXW7−/−*^ cells (Fig. 2e) and FBXW7-knockdown A549 cells (Fig. 2h) and since ERK3 bound with different cullins and FBXO subfamily members (Fig. 1d, f), we conclude that there are other E3 ligases that regulate ERK3 protein levels, resulting in different roles depending on the cellular context.

The subcellular localization events of enzymes are rate-limiting events in cellular processes and determine enzymes’ roles in responses to diverse physiological conditions^38^. To interact with binding partners, enzymes must respond to stimuli initiated by specific signaling pathways. ERK3 is a serine/threonine kinase that exhibits maximum activity at the time of production and has a half-life of <30 min^14^. Thus, we believe that regulation of the stability of ERK3 controls its activity. Mechanistic studies on ERK3 activity regulation have shown that ERK3/ERK4 heterodimer formation is required for the activation of MK5^39^ due to phosphorylation at Thr182^40^ and that this results in the translocation of ERK3 and ERK4 from the nucleus to the cytoplasm^32,40^. Furthermore, FIH- or PHD3-dependent mechanisms have been suggested to underlie the ubiquitination of ERK3 induced by its hydroxylation^41^. Furthermore, studies have suggested that among the enzymes that inhibit ERK3 hydroxylation, HUWE1 and UBE3A are closely related to ubiquitination, and RAD23b and ECM29 are related to ubiquitin recognition and proteasome degradation^41^. However, evidence regarding the ubiquitination of ERK3 indicates that its deubiquitination by ubiquitin-specific protease 20 (USP20) dose-dependently prolongs its half-life^42^. These results strongly support our hypothesis that ERK3 ubiquitination regulates ERK3 activity. However, no clear evidence has been published regarding the induction of ERK3 ubiquitination by E3 ligases. In the present study, FBXW7 was found to be a novel binding partner of ERK3 (Fig. 1a), and HCT116^*FBXW7−/−*^ cells were found to contain higher ERK3 protein levels than HCT116^*FBXW7+/+*^ cells (Fig. 2d). Notably, the ubiquitinated ERK3 protein levels were lower in HCT116^*FBXW7−/−*^ cells than in HCT116^*FBXW7+/+*^ cells (Fig. 2g). Thus, it appears that the FBXW7-ERK3 signaling axis plays an important role in the regulation of ERK3 activity in response to specific stimuli. However, the signaling pathway upstream of ERK3 has yet to be elucidated.

## Supplementary information

Supplemental Materials
