# Supplementary material for: FBXW7-mediated ERK3 degradation regulates the proliferation of lung cancer cells
Source: Exp Mol Med. 2022 Jan 12;54(1):35–46. doi: 10.1038/s12276-021-00721-9 (PMC8813941; doi:10.1038/s12276-021-00721-9)
Supplement: Supplementary file 1 — Supplemental Materials [file 12276_2021_721_MOESM1_ESM.pdf]

**Supplementary Information**

# FBXW7-mediated ERK3 degradation regulates the proliferation of lung cancer cells

Hyun-Jung An[1,2], Cheol-Jung Lee[1,3], Ga-Eun Lee[1,2], Youngwon Choi[1,2], Dohyun Jeung[1,2], Weidong Chen[1,2], Hye Suk Lee[2], Han Chang Kang[2], Joo Young Lee[2], Dae Joon Kim[4], Jin-Sung Choi[1], Eun Suh Cho[5], Jong-Soon Choi[3,6], and Yong-Yeon Cho[1,2,*]

## Contents for Supplementary information

**1. Supplementary Materials and Methods:** Transfection, Western blotting, Site-directed mutagenesis, Real-time PCR, and Foci formation assay

**2. Supplementary Data:** Supplementary Fig. 1-4

## Supplementary Materials and Methods

### Transfection

When cells reached ~50% confluence, plasmids were transfected using H4000 transfection reagent (Cat #: 4000-05, Engreen, North Shore, Auckland, New Zealand), according to the manufacturer's instructions. Briefly, a transfection mixture containing 4 μg of an expression vector was spread dropwise onto 100-mm culture dishes and cultured for 4 h. Cells were then refed with complete growth medium, cultured overnight, and harvested at 36 or 48 h after transfection. Cell lysates were then prepared and subjected to Western blotting or IP.

### Western blotting

To obtain proteins for western blotting, cells were lysed by freeze/thawing in NP-40 cell lysis buffer (150 mM NaCl, 40 mM Tris pH 8.0, and 0.5% NP-40; Roche protease inhibitor cocktail), and supernatants were recovered by centrifugation at 4°C. Protein concentrations were determined using the DC protein assay kit (Bio-Rad Laboratories, Hercules, CA, USA). Equal amounts of proteins were resolved by 10–15% SDS-PAGE and transferred to polyvinylidene difluoride (PVDF) membranes (Merck Millipore Ltd., Burlington, MA, USA). Membranes were then blocked with 5% skim milk/1× PBS containing 0.05% Tween 20 (PBS-T) and hybridized with specific antibodies. Western blots were visualized using an enhanced chemiluminescence detection system (Amersham Biosciences, Piscataway, NJ, USA) and a Chemidoc XRS imager system (Bio-Rad Laboratories).

### Site-directed mutagenesis

Plasmid pCMV-myc-ERK3 was used to generate the point mutants pCMV-myc-ERK3-T417A/T421A, S428A/E432A, T448A/S452A, and S477A/E481A in the C34D of ERK3 using a site-directed mutagenesis kit (QuikChange II site-directed mutagenesis kit, Cat #: 200524, Agilent Technologies, Santa Clara, CA, USA). The primers used to introduce mutations were designed and synthesized by Macrogen Inc. (Geumcheon-gu, Seoul) and were as follows; T417A/T421A, 5′-GCT TTT GAT GCC AAT

TAC TCT GCC GAG CCT TG-3′; S428A/E432A, 5′-GTT GGC AAT ACG CCG ATC ATC ATG CCA ACA AAT ATT G-3′; T448A/S452A, 5′-GTA ACT ACA AAG CCA GGT CAT CAG CCT ATT TAG ATA AC-3′; and S477A/E481A, 5′-GAT CTT GCC AAT TGG AAA GCC CAA AGC AAA G-3′. The procedure used for site-directed mutagenesis was based on a PCR-based strategy according to the manufacturer's instructions. Template DNA was destroyed using Dpn I (Cat #: EBR-1045, Elpisbio, Seo-gu, Daejeon, Korea). The accuracies of mutant constructs were confirmed by DNA sequencing (Macrogen Inc.).

**Real-time polymerase Chain reaction**

HCT116$^{FBXW7+/+}$ and HCT116$^{FBXW7-/-}$ ($5\times10^5$) cells were seeded into 60-mm dishes and cultured. When cells reached ~90% confluence they were harvested, and total RNAs were isolated using Trizol (Invitrogen, Carlsbad, CA, USA). ERK3 gene expression levels were quantified by real-time PCR using an ERK3 specific real-time primer set (Cat #: 4331182; Hs00833126_g1, Taqman), a GAPDH (glyceraldehyde 3-phosphate dehydrogenase) specific real-time primer set (Cat #: 4331182; Hs02786624_g1, Taqman), and a TaqMan RNA-to-CT 1-step kit (Applied Biosystems, Foster City, CA, USA), according to the manufacturers' instructions. Ct values of ERK3 were normalized versus GAPDH (the internal control).

**Foci formation assay**

A549 ($5\times10^3$ cells/well) and H1299 ($5\times10^3$ cells/well) cells stably expressing sh-ERK3 or sh-FBXW7 were seeded in 6-well culture plates in complete media and cultured at 37°C in a 5% $CO_2$ incubator. The medium was changed every three days until foci grew to an ap-propriate size. Foci formed were stained with 0.5% crystal violet for 20 min. Cells were then washed 3 times with 1× PBS, and colony numbers were counted using Image J (ver. 1.46r).

**2. Supplementary Data**

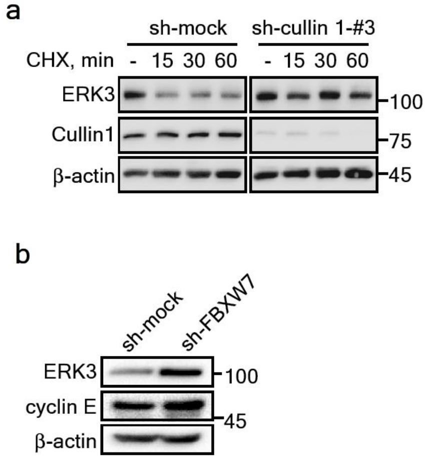


**Supplementary Fig. 1 Involvement of cullin1 and FBXW7 for ERK3 protein stability regulation. a** Cullin 1 knockdown attenuates ERK3 turnover in A549 cells. A549 lung cancer cells stably infected with Lenti-sh-cullin 1 viral particle were treated with CHX (10 μg/ml), harvested at indicated time point, and then used to prepare cell lysates. The cell lysates (30 μg) were used to visualize ERK3 protein levels by Western blotting. **b** FBXW7 knockdown increases ERK3 protein in A549 cells. Cell lysates (30 μg/lane) obtained from A549 lung cancer cells stably infected with Lenti-sh-FBXW7 viral particle were subjected to evaluate ERK3 protein level by Western blotting. Cyclin E was used for internal control for FBXW7 knockdown effects. **a-b** β-actin was used as the internal control to ensure equal protein loading.

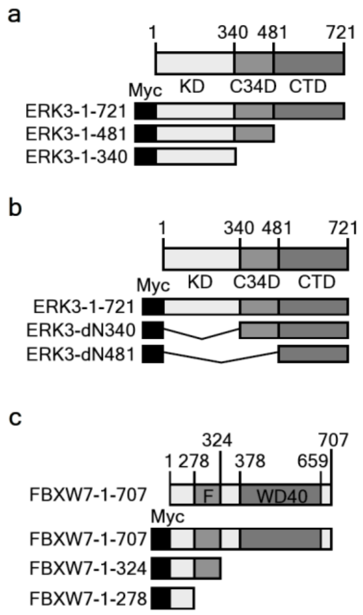


**Supplementary Fig. 2 Construction maps of ERK3 and FBXW7 deletion expression vectors. a** Construction maps of ERK3 deletion expression vectors. The expression constructs were designed by serial deletion from C-terminus of ERK3 proteins. **b** Construction maps of ERK3 deletion expression vectors. The expression constructs were designed by serial deletion from N-terminus of ERK3 proteins. **c** Construction maps of FBXW7 deletion expression vectors. The expression constructs were designed by serial deletion from N-terminus of FBXW7 proteins. **a-c** Numbers in domain structures of ERK3 or FBXW7 indicate amino acid number.

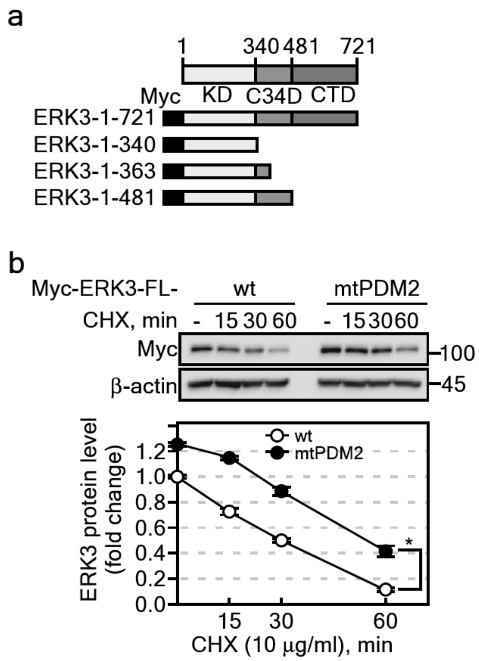


**Supplementary Fig. 3 T417/T421 of ERK3 acts as a degron motif of FBXW7. a** Construction maps of C34D domain deletion mutants of ERK3. **b** Protein turnover of ERK3-mtPDM2 (T417A/T421A) mutants were compared to ERK3-wt at indicated time points after CHX (10 μg/ml). Myc-ERK3 proteins were visualized by Western blotting (30 μg/lane) using Myc antibody. The band intensities were measured and normalized by densitometry using NIH Image J (ver.1.52a) computer program.

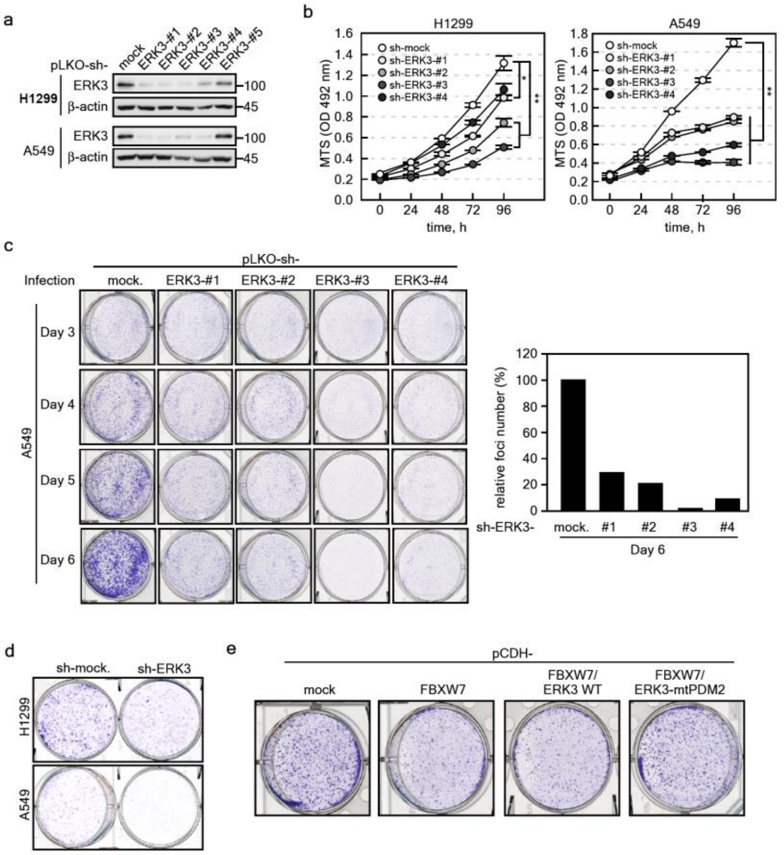


**Supplementary Fig. 4 The FBXW7-ERK3 axis regulates the proliferation of lung cancer cells. a** Knockdown efficiency of pLKO-sh-ERK3 clones. #1 to #5 indicates different sh-RNA clones. **b** Knockdown effects of pLKO-sh-ERK3 clones on cell proliferation. H1229 and A549 cells stably infected with each different clone of lenti-sh-ERK3 viral particles were seeded into 96-well plates. The cell proliferation was measured at each indicated time points by MTS assay. **c** Knockdown effects of pLKO-sh-ERK3 clones on focus formation. A549 cells in (**b**) were seeded at $5\times10^3$ cells/well in 6-well plates, cultured for 3-6 days, and stained with 0.5% crystal violet. **d** ERK3 knockdown effects of foci formation in H1299 and A549 cells. H1299 or A549 cells in (**b**) were seeded at $5\times10^3$ cells/well in 6-well plates, cultured for 4 days, and stained with 0.5% crystal violet. Colony numbers were then counted using Image J (ver.1.52a). Relative colonies numbers are shown in **Fig. 5a** in the main text. **e** ERK3-mtPDM2 played an important role in ERK3-stability-mediated lung cancer cell proliferation. Lentiviral overexpression vectors including ERK3-wt, FBXW7, and ERK3-mtPDM2 were infected as indicated. The cells were assessed for foci

formation as described in (**b**). Colony numbers were counted using Image J (ver.1.52a). Relative colonies numbers are provided in **Fig. 5d** in the main text.
